# Comparison of tillage costs among eight paddy farm regions in East Kalimantan, Indonesia

**DOI:** 10.12688/f1000research.16991.1

**Published:** 2018-12-19

**Authors:** Karmini Karmini

**Affiliations:** 1 Faculty of Agriculture, University of Mulawarman, City of Samarinda, Province of East Kalimantan, 75123, Indonesia

**Keywords:** Cost, East Kalimantan, land, paddy farm, tillage.

## Abstract

**Background:** Tillage is done to prepare land for wetland paddy farming, and it is commonly done by hand tractor. The purposes of this study were to identify the levels of ownership of hand tractor by paddy farmers, to describe the rental of hand tractor in rural areas, to calculate and compare the tillage costs on eight paddy farm regions, and to understand the utilization of farm machinery for paddy farming in East Kalimantan, Indonesia.

**Methods:** The study areas were Subcities/Subdistricts of North Bontang, South Bontang, Muara Muntai, Loa Janan, Tenggarong Seberang, Waru, Penajam, and Babulu. Data collection was done by interviewing 380 respondents. Analysis of data used the Chi Square test.

**Results:** The number of hand tractor renters (87.37%) in East Kalimantan 2014 was bigger than that of hand tractor owners (12.63%). The tillage costs in Tenggarong Seberang, Loa Janan, and Muara Muntai in 2014 were IDR700,000.00 ha ^-1^, IDR750,000.00 ha ^-1^, and IDR700,000.00 ha ^-1^, respectively. Tillage costs were the same in Babulu, Penajam, Waru, South Bontang, and North Bontang (IDR1,000,000.00  ha ^-1^ in each district).

**Conclusions:** There are significant differences the number of hand tractor owners, the number of hand tractor renters, and the tillage costs among the eight paddy farm regions in East Kalimantan, Indonesia.

## Introduction

Wetland paddy farming in East Kalimantan is a method of modern farming in which paddy farmers commonly by using hand tractor in land preparation. Tillage cost has important role in cost structure of paddy farming. The tillage cost can vary significantly, and negatively affects paddy farm income in East Kalimantan, Indonesia ( Karmini, 2017). The increase of tillage cost leads the increase of production cost of paddy farming and the decrease of paddy farm income and household income of paddy farmers. This is supported by the research result of Larson & Plessmann (2009).

Wetland paddy farming is done in most regions in East Kalimantan. Information is needed about the tillage cost in different paddy farm areas to formulate policy on farm machinery utilization in specific areas containing paddy farms. The purposes of this study were to identify the ownership of hand tractor by paddy farmers, to describe the rental of hand tractors in rural areas, to calculate and compare the tillage costs on eight paddy farm regions, and to know the machinery utilization for paddy farming in East Kalimantan, Indonesia. The hypotheses of this study were that there are no significant differences the number of hand tractor owners, the number of hand tractor renters, and the tillage costs among the eight paddy farm regions in East Kalimantan, Indonesia.

## Methods

### Study area

This study was held from November 2013 to April 2014 in Province of East Kalimantan, Republic of Indonesia. The determination of study areas based on two-stage clustered sampling. The study areas were Bontang City (North Bontang and South Bontang), Kutai Kartanegara District (Muara Muntai, Loa Janan, and Tenggarong Seberang), and Penajam Paser Utara District (Waru, Penajam, and Babulu).

### Subject recruitment

The overall population in the areas examined in this study was 36,970 households of paddy farmers. The minimum sample size for populations of 20,000 and 50,000 people is 377 and 382, respectively ( Rea & Parker, 1997). The sample size used in this study was therefore 380 respondents. The determination of the number of respondents in each study areas was based on proportional sampling.

Purposive sampling was applied in selecting respondents. Inclusion criterias for respondents were farmers who are currently engaged in wetland paddy farming, lived minimum 5 years in study area, and had experience continuously minimum 2 years cultivate paddy. Exclusion criterias for respondents were lived less than 5 years in study area and had experience less than 2 years cultivate paddy but farmers have own land or become labors. Respondents were paddy farmers who are currently engaged in wetland paddy farming. The researcher went to paddy field and met with potential respondents in person, then provided information on the purposes of study and the right of them to not answer the questions at any time and assured that the data would be kept confidential and only aggregate data would be used. After they gave the consent to be interviewed, they were given the choice to decide the place for the interviews using the questionnaire ( Karmini, 2018), either at home or other places which were convenient for them. Both the researcher and respondents discussed directly at the same place. This study was approved by Head of Department of Agribusiness, Faculty of Agriculture, University of Mulawarman (Tetty Wijayanti, SP, MP; approval number 2104/UN17.3/TU/2013). Each participant gave their written informed consent to participate in the study.

### Statistical analysis

This study performed analysis by using the software of IBM SPSS Statistics 20 and tested hypotheses using the Chi Square test with α = 0.05.

## Results and discussion

### Characteristics of respondents

All 380 respondents completed the questionnaire in full. From our study, we observed that paddy farmers in East Kalimantan, Indonesia, are typically male, married, 3–4 members in their household, and are Javanese.

### Hand tractor ownership

 A small number of paddy households had the ability to buy hand tractor in the study areas (12.63%) ( Table 1). There are significant differences the number of hand tractor owners among the eight paddy farm regions in East Kalimantan, Indonesia (χ ^2^_calculated_ 62.35 > χ ^2^_ table dk = 7; α = 0,05 _14.1). Narayanamoorthy *et al*. (2014) found that the factors such as coverage of irrigation, yield enhancing inputs cost, land-labor ratio, and human labor use in man-hours have significanlty influence the use of machine labor in paddy cultivation.

**Table 1.** Number of respondents who were hand tractor owners in East Kalimantan the year 2014.

City/District	Region (Subcity/Subdistrict)	Respondent (paddy household)	Percentage (%)	Hand tractor owner (respondent)	Percentage (%)
Bontang City	North Bontang	1	0.26	0	0.00
	South Bontang	2	0.53	0	0.00
Kutai Kartanegara Regency	Muara Muntai	4	1.05	0	0.00
	Loa Janan	17	4.47	2	0.53
	Tenggarong Seberang	128	33.68	17	4.47
Penajam Paser Utara Regency	Waru	16	4.21	4	1.05
	Penajam	84	22.11	17	4.47
	Babulu	128	33.68	8	2.11
Total		380	100	48	12.63

### Hand tractor renters

 Farmers who did not own a hand tractor (87.37%) could rent from the owners of hand tractor who live in the same village or from nearby village ( Table 2). There are significant differences the number of hand tractor renters among the eight paddy farm regions in East Kalimantan, Indonesia (χ ^2^_calculated_ 429.45 > χ ^2^_table dk = 7; α = 0,05 _14.1). This was reasonable, because owning a hand tractor is very costly. Hand tractor prices ranged from IDR6,000,000.00 each ^-1^ to IDR25,000,000.00 each ^-1^. 

**Table 2.** Number of respondents who were hand tractor renters in East Kalimantan the year 2014.

City/District	Region (Subcity/Subdistrict)	Respondent (paddy household)	Percentage (%)	Hand tractor owner (respondent)	Percentage (%)
Bontang City	North Bontang	1	0.26	1	0.26
	South Bontang	2	0.53	2	0.53
Kutai Kartanegara Regency	Muara Muntai	4	1.05	4	1.05
	Loa Janan	17	4.47	15	3.95
	Tenggarong Seberang	128	33.68	111	29.21
Penajam Paser Utara Regency	Waru	16	4.21	12	3.16
	Penajam	84	22.11	67	17.63
	Babulu	128	33.68	120	31.58
Total		380	100	332	87.37

### Tillage cost

Farm size varies among paddy farming households in all regions (0.25-5.00 ha). Small land-holding farmers in the study areas did not have constraints to rent and use of hand tractors because the wetland fields for the most part are located in same area. However, Hristova & Maddock (1993) mentioned that land size could be a constraint in applying mechanized farming. The proportion of machine labour costs (11.13%) of total cost of cultivation of borewell irrigated paddy in Tumakuru District, India ( Hamsa *et al*., 2017).

The tillage costs ( Table 3) in Tenggarong Seberang and Muara Muntai were lower than those in Loa Janan. Tillage costs were same in other five regions. Limitations of the study, such as limited access to several study areas which more time was needed to collect data, influenced the diversity of respondents and data. There are significant differences the tillage costs among the eight paddy farm regions in East Kalimantan, Indonesia (χ ^2^_calculated_ 17.01 > χ ^2^_ table dk = 7; α = 0,05 _14.1). The difference of tillage costs could be happened because of the difference of buying price of machine, operator wage, and machine maintenance cost.

**Table 3.** Number of respondents and mean farm sizes and tillage costs in East Kalimantan the year 2014.

City/District	Region (Subcity/Subdistrict)	Respondent (paddy household)	Mean of farm size (ha)	Mean of tillage cost (IDR)	Mean of tillage cost (IDR ha ^-1^)
Bontang City	North Bontang	1	0.63	625,000.00	1,000,000.00
	South Bontang	2	0.63	625,000.00	1,000,000.00
Kutai Kartanegara Regency	Muara Muntai	4	0.75	525,000.00	700,000.00
	Loa Janan	17	0.71	529,411.76	750,000.00
	Tenggarong Seberang	128	1.08	757,421.88	700,000.00
Penajam Paser Utara Regency	Waru	16	1.19	1,187,500.00	1,000,000.00
	Penajam	84	1.02	1,020,833.33	1,000,000.00
	Babulu	128	1.76	1,761,328.13	1,000,000.00
Total		380			

### Farm machinery 

Hand tractor usage is still recommended for the development of paddy farming as an important physical asset in paddy farming. The number of hand tractors in rural areas could be increased, either through purchase by paddy farmers or by grants from government, to decrease the tillage cost and production cost, thus increasing income of paddy farming and paddy farmers.

## Conclusions

The number of paddy households as hand tractor owners and hand tractor renters in East Kalimantan in 2014 were 12.63% and 87.37%, respectively. The tillage cost was between IDR700,000.00 ha ^-1^ and IDR1,000,000.00 ha ^-1^. There are significant differences the number of hand tractor owners, the number of hand tractor renters, and the tillage costs among the eight paddy farm regions in East Kalimantan, Indonesia.

## Data availability

### Underlying data

The answers to the questionnaire, along with basic demographic information generated in this study are available on OSF. DOI: https://doi.org/10.17605/OSF.IO/C7EX9 ( Karmini, 2018).

### Extended data

The questionnaire used in this study is available on OSF. DOI: https://doi.org/10.17605/OSF.IO/C7EX9 ( Karmini, 2018).

Data are available under the terms of the Creative Commons Zero "No rights reserved" data waiver (CC0 1.0 Public domain dedication).
