# Comparison of tillage costs among eight paddy farm regions in East Kalimantan, Indonesia

**DOI:** 10.12688/f1000research.16991.2

**Published:** 2021-05-07

**Authors:** Karmini Karmini, Karyati Karyati

**Affiliations:** 1 Faculty of Agriculture, University of Mulawarman, City of Samarinda, Province of East Kalimantan, Indonesia; 2 Faculty of Forestry, University of Mulawarman, , City of Samarinda, Province of East Kalimantan, Indonesia

**Keywords:** Cost, East Kalimantan, land, paddy farm, tillage.

## Abstract

**Background:** Tillage is done to prepare land for wetland paddy farming, and it is commonly done by hand tractor. The purposes of this study were to identify the levels of ownership of hand tractor by paddy farmers, to describe the rental of hand tractor in rural areas, to calculate and compare the tillage costs on eight paddy farm regions, and to understand the utilization of farm machinery for paddy farming in East Kalimantan, Indonesia.

**Methods:** The study areas were Subcities/Subregencies of North Bontang, South Bontang, Muara Muntai, Loa Janan, Tenggarong Seberang, Waru, Penajam, and Babulu. Data collection was done by interviewing 380 respondents. Analysis of data used descriptive statistics, the Chi Square One Sample test, and One Way Anova.

**Results:** The number of hand tractor renters (87.37%) in East Kalimantan 2014 was bigger than that of hand tractor owners (12.63%). The tillage costs in Tenggarong Seberang, Loa Janan, and Muara Muntai in 2014 were USD48.56 ha ^-1^, USD52.03 ha ^-1^, and USD48.56 ha ^-1^, respectively. Tillage costs were the same in Babulu, Penajam, Waru, South Bontang, and North Bontang (USD69.37 ha ^-1^ in each regency).

**Conclusions:** There are very significant differences the number of hand tractor owners, the number of hand tractor renters, and the tillage costs among some regions in East Kalimantan, Indonesia. Farm machinery is needed in development of paddy farming.

## Introduction

Wetland paddy farming in East Kalimantan is a method of modern farming in which paddy farmers commonly by using hand tractor in land preparation. Tillage cost has important role in cost structure of paddy farming. The tillage cost can vary significantly, and negatively affects paddy farm income in East Kalimantan, Indonesia ( Karmini, 2017). The increase of tillage cost leads the increase of production cost of paddy farming and the decrease of paddy farm income and household income of paddy farmers. This is supported by the research result of Larson & Plessmann (2009).

Wetland paddy farming is done in most regions in East Kalimantan. Information is needed about the tillage cost in different paddy farm areas to formulate policy on farm machinery utilization in specific areas containing paddy farms. The purposes of this study were to identify the ownership of hand tractor by paddy farmers, to describe the rental of hand tractors in rural areas, to calculate and compare the tillage costs on eight paddy farm regions, and to know the machinery utilization for paddy farming in East Kalimantan, Indonesia. The hypotheses of this study were that there are no significant differences the number of hand tractor owners, the number of hand tractor renters, and the tillage costs among the eight paddy farm regions in East Kalimantan, Indonesia.

## Methods

### Study area

This study was held from November 2013 to April 2014 in Province of East Kalimantan, Republic of Indonesia. Paddy farmer income in East Kalimantan lower than that in West Kalimantan and South Kalimantan ( Statistics Indonesia, 2008) and paddy productivity in East Kalimantan lower than that in South Kalimantan and the average in Indonesia ( Statistics East Kalimantan, 2009). The determination of study areas based on two-stage clustered sampling. The study areas were Bontang City (North Bontang and South Bontang), Kutai Kartanegara Regency (Muara Muntai, Loa Janan, and Tenggarong Seberang), and Penajam Paser Utara Regency (Waru, Penajam, and Babulu).

### Subject recruitment

The overall population in the areas examined in this study was 36,970 households of paddy farmers. The minimum sample size for populations of 20,000 and 50,000 people is 377 and 382, respectively ( Rea & Parker, 1997). The sample size used in this study was therefore 380 respondents. The determination of the number of samples in each study areas was based on proportional sampling. Based on data of the number of paddy household each region ( Statistics East Kalimantan, 2009) multiplied by the total sample then divided by total paddy household in eight regions, it was known the sample size in North Bontang (24; 1), South Bontang (120; 2), Muara Muntai (206; 4), Loa Janan (1,002; 17), Tenggarong Seberang (7,388; 128), Waru (908; 16), Penajam (4,829; 84), and Babulu (7,343; 128).

Random sampling was applied in selecting samples. Farmers who met the inclusion and exclusion criterias had equal probability to be chosen as samples. Inclusion criterias for samples were farmers who are currently engaged in wetland paddy farming, lived minimum 5 years in study area, and had experience continuously minimum 2 years cultivate paddy. Exclusion criterias for samples were lived less than 5 years in study area and had experience less than 2 years cultivate paddy but farmers have own land or become labors. The random number method assigned every sample a number, then randomly picked samples by using a table of random numbers. The researchers employed the enumerators to help the interviews process. The researchers and enumerators went to paddy field and met with potential respondents in person, then provided information on the purposes of study and the right of them to not answer the questions at any time and assured that the data would be kept confidential and only aggregate data would be used. After they gave the consent to be interviewed, they were given the choice to decide the place for the interviews using the questionnaire ( Karmini, 2018), either at home or other places which were convenient for them. The researchers, enumerators, and respondents discussed directly at the same place. This study was approved by Head of Department of Agribusiness, Faculty of Agriculture, University of Mulawarman (Tetty Wijayanti, SP, MP; approval number 2104/UN17.3/TU/2013). Each participant gave their written informed consent to participate in the study.

### Statistical analysis

This study performed analysis by using the software of IBM SPSS Statistics 24. Descriptive statistics was applied to count frequency, total, percentage, maximum, and minmum. Tested hypotheses used the Chi Square One Sample and One Way Anova with α = 0.01 and Pobhirun & Pinitsoontorn (2019) and Ogunbanwo *et al.* (2019) reported the socio-demografic data by using descriptive statistics and analysed hypotheses with Chi Square test. The exchange rate of 1 USD was 14,415.25 IDR on 28 March 2021.

## Results and discussion

### Characteristics of respondents

All 380 respondents completed the questionnaire in full (Extended data). From our study, we observed that the most of paddy farmers in East Kalimantan, Indonesia, are typically male (95.53%), married (93.42%), 4–6 members in their household (52.63%), and are Javanese (93.16%), have 1-2 ha paddy farm size (58.95%), handtractor renter (87.37%), buying price of handtractor between USD1.001,00 – USD1.500,00 unit ^-1^ (7.63%), and rent cost of handtractor between USD51.00 – USD100.00 (33.42%) ( Table 1 and Underlying data).

**Table 1.** Respondents' socioeconomic-demographic characteristics.

No.	Characteristics	Level/Category	Frequency (respondent)								Total	Precentage (%)
			Bontang City		Kutai Kartanegara Regency			Penajam Paser Utara Regency				
			North Bontang	South Bontang	Muara Muntai	Loa Janan	Tenggarong Seberang	Waru	Penajam	Babulu		
1	Residence	Subcity/subregency	1	2	4	17	128	16	84	128	380	100.00
2	Gender	Male	1	2	4	16	128	13	79	120	363	95.53
		Female	-	-	-	1	-	3	5	8	17	4.47
3	Marital status	Married	1	2	4	17	125	15	79	112	355	93.42
		Single	-	-	-	-	3	-	-	-	3	0.79
		Divorced	-	-	-	-	-	-	3	9	12	3.16
		Widow	-	-	-	-	-	1	2	7	10	2.63
4	Members of household	1–3 person	1	-	-	8	51	3	33	76	172	45.26
		4–6 person	-	2	3	9	77	10	47	52	200	52.63
		7–9 person	-	-	1	-	-	2	4	-	7	1.84
		10 person	-	-	-	-	-	1	-	-	1	0.26
5	Ethnicity	Javanese	-	-	-	17	122	14	82	119	354	93.16
		Sundanese	-	-	-	-	-	-	-	7	7	1.84
		Kutainese	-	-	3	-	3	-	-	-	6	1.58
		Banjarnese	-	-	1	-	-	1	-	2	4	1.05
		Bugisnese	1	2	-	-	3	1	2	-	9	2.37
6	Paddy farm size	< 1,00 ha	1	1	3	9	54	5	38	10	121	31.84
		1,00–2,00 ha	-	1	1	8	69	8	43	94	224	58.95
		2,10–3,00 ha	-	-	-	-	5	3	2	16	26	6.84
		> 3,00 ha	-	-	-	-	-	-	1	8	9	2.37
7	Handtractor ownership	Owner	-	-	-	2	17	4	17	8	48	12.63
		Renter	1	2	4	15	111	12	67	120	332	87.37
8	Buying price of handtractor	<USD500.00 unit ^-1^	-	-	-	-	-	-	-	1	1	0.26
		USD501.00 - USD1,000.00 unit ^-1^	-	-	-	2	11	-	3	-	16	4.21
		USD1.001.00 - USD1,500.00 unit ^-1^	-	-	-	-	6	3	13	7	29	7.63
		USD1,501.00 - USD2,000.00 unit ^-1^	-	-	-	-	-	1	1	-	2	0.53
9	Rent cost of handtractor	< USD50.00	1	1	3	9	74	5	27	5	125	32.89
		USD51.00 - USD100.00	-	1	1	6	32	6	29	52	127	33.42
		USD101.00 – USD150.00	-	-	-	-	5	-	8	44	57	15.00
		>USD150.00	-	-	-	-	-	1	3	19	23	6.05

### Hand tractor ownership

A small number of paddy households had the ability to buy hand tractor in the study areas (12.63%) ( Table 2). There are very significant differences the number of hand tractor owners among the five paddy farm regions (Loa Janan, Tenggarong Seberang, Waru, Penajam, and Babulu) in East Kalimantan, Indonesia (χ ^2^_alculated_ = 20.96 > χ ^2^_ table df = 4; α = 0.01_ = 13.28; *р*-value = 0.0003 < α = 0.01). Narayanamoorthy *et al*. (2014) found that the factors such as coverage of irrigation, yield enhancing inputs cost, land-labor ratio, and human labor use in man-hours have significanlty influence the use of machine labor in paddy cultivation.

**Table 2.** Number of respondents who were hand tractor owners in East Kalimantan the year 2014.

City/Regency	Region (Subcity/Subregency)	Respondent (paddy household)	Percentage (%)	Hand tractor owner (respondent)	Percentage (%)
Bontang	North Bontang	1	0.26	0	0.00
	South Bontang	2	0.53	0	0.00
Kutai Kartanegara	Muara Muntai	4	1.05	0	0.00
	Loa Janan	17	4.47	2	0.53
	Tenggarong Seberang	128	33.68	17	4.47
Penajam Paser Utara	Waru	16	4.21	4	1.05
	Penajam	84	22.11	17	4.47
	Babulu	128	33.68	8	2.11
Total		380	100	48	12.63

### Hand tractor renters

 Farmers who did not own a hand tractor (87.37%) could rent from the owners of hand tractor who live in the same village or from nearby village ( Table 3). There are very significant differences the number of hand tractor renters among the eight paddy farm regions in East Kalimantan, Indonesia (χ ^2^_calculated_ = 429.45 > χ ^2^_table df = 7; α = 0.01_ = 18.48; *p*-value = 1.15×10 ^-88^ < α = 0.01). This was reasonable, because owning a hand tractor is very costly. Hand tractor prices ranged from USD 416.23 unit ^-1^ to USD1,734.27 unit ^-1^. 

**Table 3.** Number of respondents who were hand tractor renters in East Kalimantan the year 2014.

City/Regency	Region (Subcity/Subregency)	Respondent (paddy household)	Percentage (%)	Hand tractor renter (respondent)	Percentage (%)
Bontang	North Bontang	1	0.26	1	0.26
	South Bontang	2	0.53	2	0.53
Kutai Kartanegara	Muara Muntai	4	1.05	4	1.05
	Loa Janan	17	4.47	15	3.95
	Tenggarong Seberang	128	33.68	111	29.21
Penajam Paser Utara	Waru	16	4.21	12	3.16
	Penajam	84	22.11	67	17.63
	Babulu	128	33.68	120	31.58
Total		380	100	332	87.37

### Tillage cost

Farm size varies among paddy farming households in all regions (0.25-5.00 ha). Small land-holding farmers in the study areas did not have constraints to rent and use of hand tractors because the wetland fields for the most part are located in same area. However, Hristova & Maddock (1993) mentioned that land size could be a constraint in applying mechanized farming. The proportion of machine labour costs (11.13%) of total cost of cultivation of borewell irrigated paddy in Tumakuru District, India ( Hamsa *et al*., 2017).

The tillage costs ( Table 4) in Tenggarong Seberang and Muara Muntai were lower than those in Loa Janan. Tillage costs were same in other five regions. The amount of hand tractor rental cost at the research location includes machine rental cost, machine maintenance cost, and operator wage. Limitations of the study, such as limited access to several study areas which more time was needed to collect data, influenced the diversity of respondents and data. There are very significant differences the tillage costs among Bontang, Kutai Kartanegara, and Penajam Paser Utara in East Kalimantan, Indonesia ( *F*_calculated_ = 39,236.54 > *F*_ table dfn = 2; dfd = 377; α = 0.01 _ = 8.88; *p*-value = 0.00 < α = 0.01). The difference of tillage costs could be happened because of the difference of buying price of machine, operator wage, and machine maintenance cost.

**Table 4.** Number of respondents and mean farm sizes and tillage costs in East Kalimantan the year 2014.

City/Regency	Region (Subcity/Subregency)	Respondent (paddy household)	Mean of farm size (ha)	Mean of tillage cost (USD)	Mean of tillage cost (USD ha ^-1^)
Bontang	North Bontang	1	0.63	43.36	69.37
	South Bontang	2	0.63	43.36	69.37
Kutai Kartanegara	Muara Muntai	4	0.75	36.42	48.56
	Loa Janan	17	0.71	36.73	52.03
	Tenggarong Seberang	128	1.08	52.54	48.56
Penajam Paser Utara	Waru	16	1.19	82.38	69.37
	Penajam	84	1.02	70.82	69.37
	Babulu	128	1.76	122.19	69.37
Total		380			

### Farm machinery 

Hand tractor usage is still recommended for the development of paddy farming as an important physical asset in paddy farming. The number of hand tractors in rural areas could be increased, either through purchase by paddy farmers or by grants from government, to decrease the tillage cost and production cost, thus increasing income of paddy farming and paddy farmers.

## Conclusions

The number of paddy households as hand tractor owners and hand tractor renters in East Kalimantan in 2014 were 12.63% and 87.37%, respectively. The tillage cost was between USD48.56 ha ^-1^ and USD69.37 ha ^-1^. There are very significant differences the number of hand tractor owners among Loa Janan, Tenggarong Seberang, Waru, Penajam, and Babulu and the number of hand tractor renters among the eight paddy farm regions in East Kalimantan, Indonesia. There are very significant differences the tillage costs among Bontang, Kutai Kartanegara, and Penajam Paser Utara in East Kalimantan, Indonesia. Those might be caused the difference of paddy farmers number, labor availability, farming costs, farmer prosperity, land ownership, paddy farm size, hand tractor price, operator wage, and hand tractor maintenance cost among regions. Farm machinery is required to develop the paddy farming. Bontang and Penajam Paser Utara are the recommended regions for the activities in relation to increase the ownership of hand tractors because have higher tillage costs rather than Kutai Kartanegara. 

## Data availability

### Underlying data

The answers to the questionnaire, along with basic demographic information generated in this study are available on OSF. DOI: https://doi.org/10.17605/OSF.IO/C7EX9 ( Karmini, 2018).

### Extended data

The questionnaire used in this study is available on OSF. DOI: https://doi.org/10.17605/OSF.IO/C7EX9 ( Karmini, 2018).

Data are available under the terms of the Creative Commons Zero "No rights reserved" data waiver (CC0 1.0 Public domain dedication).
