# Supplementary material for: Comparison of tillage costs among eight paddy farm regions in East Kalimantan, Indonesia
Source: F1000Res. 2021 May 7;7:1951. Originally published 2018 Dec 19. [Version 2] doi: 10.12688/f1000research.16991.2 (PMC8256191; doi:10.12688/f1000research.16991.2)
Supplement: Peer review history

# Peer review: Reviewer response for version 2

**DOI:** 10.5256/f1000research.56051.r84802

**Published:** 2021-07-02

**Authors:** Ganganee Chandima Samaraweera

**Affiliations:** 1 Department of Agricultural Economics , Faculty of Agriculture, University of Ruhuna, Kamburupitiya, Matara, Sri Lanka

I read the revised version. Now it’s OK from my side.

Is the work clearly and accurately presented and does it cite the current literature?

No

If applicable, is the statistical analysis and its interpretation appropriate?

Partly

Are all the source data underlying the results available to ensure full reproducibility?

Partly

Is the study design appropriate and is the work technically sound?

Partly

Are the conclusions drawn adequately supported by the results?

Partly

Are sufficient details of methods and analysis provided to allow replication by others?

Partly

Reviewer Expertise:

Agricultural Economics, Agricultural Marketing, Consumer behavior

I confirm that I have read this submission and believe that I have an appropriate level of expertise to confirm that it is of an acceptable scientific standard.

# Peer review: Reviewer response for version 1

**DOI:** 10.5256/f1000research.18576.r78026

**Published:** 2021-03-11

**Authors:** Ganganee Chandima Samaraweera

**Affiliations:** 1 Department of Agricultural Economics , Faculty of Agriculture, University of Ruhuna, Kamburupitiya, Matara, Sri Lanka

Even the author mentioned significant differences, p values are not given (specially in case of ownership and other variables, it says, (There are significant differences the number of hand tractor owners, the number of hand tractor renters, and the tillage costs among the eight paddy farm regions in East Kalimantan, Indonesia.). However, it didn't show clearly.

 The demographic status of the respondents is not mentioned, and the statistical analyses are not adequate as the author used only Chi-Square test. It is a serious issue as this is the only stat method used here. Better to expand the analytical tools as this cannot be generalized to the vast majority.

 More recent literature is needed to be addressed and authors should mention the compatibility of their research findings with other relevant literature documented so far.

 In the Methodology, it should be clearly mention the  reason of selecting study areas, and the clear way of selecting the sample from the population

 Hence, I will give my recommendation after adding those comments.

Is the work clearly and accurately presented and does it cite the current literature?

No

If applicable, is the statistical analysis and its interpretation appropriate?

Partly

Are all the source data underlying the results available to ensure full reproducibility?

Partly

Is the study design appropriate and is the work technically sound?

Partly

Are the conclusions drawn adequately supported by the results?

Partly

Are sufficient details of methods and analysis provided to allow replication by others?

Partly

Reviewer Expertise:

Agricultural Economics, Agricultural Marketing, Consumer behavior

I confirm that I have read this submission and believe that I have an appropriate level of expertise to confirm that it is of an acceptable scientific standard, however I have significant reservations, as outlined above.

# Response

**Published:** 2021-04-10

**Authors:** Karmini Karmini

**Affiliations:** University of Mulawarman, Indonesia

**The authors revision for review on 11 March 2021 by Ganganee Chandima Samaraweera, Department of Agricultural Economics, Faculty of Agriculture, University of Ruhuna, Kamburupitiya, Matara, Sri Lanka.**

 Reviewer: Even the author mentioned significant differences, p values are not given (specially in case of ownership and other variables, it says, (There are significant differences the number of hand tractor owners, the number of hand tractor renters, and the tillage costs among the eight paddy farm regions in East Kalimantan, Indonesia.). However, it didn't show clearly.

 Authors: We added *р*-values in sections of Hand tractor ownership, Hand tractor renter, and Tillage cost.

 Reviewer: The demographic status of the respondents is not mentioned,

 Authors: We added Table 1 and information of respondents’ socioeconomic-demographic characteristics in section of Characteristics of respondents.

 Reviewer: and the statistical analyses are not adequate as the author used only Chi-Square test. It is a serious issue as this is the only stat method used here. Better to expand the analytical tools as this cannot be generalized to the vast majority.

 Authors: We added descriptive statistics to count the frequency, total, percentage, maximum, and minimum in section Statistical analysis. We revised Chi Square One Sample with One Way Anova to test hypothesis that there are no significant differences the tillage costs among Bontang, Kutai Kartanegara, and Penajam Paser Utara in sections of Statistical analysis and Tillage cost.

 Reviewer: More recent literature is needed to be addressed

 Authors: We added references of Pobhirun and Pinitsoontorn (2019), Statistics Indonesia (2008), Statistics East Kalimantan (2009), and Ogunbanwo et al. (2019) in section of References.

 Reviewer: and authors should mention the compatibility of their research findings with other relevant literature documented so far.

 Authors: Statistics Indonesia (2008) and Statistics East Kalimantan (2009) reported data of paddy farm income and paddy productivity in section Study area. Ogunbanwo et al. (2019) and Pobhirun and Pinitsoontorn (2019)  used descriptive statistics  and Chi Square test in their researches in section Statistical analysis.

 Reviewer: In the Methodology, it should be clearly mention the  reason of selecting study areas,

 Authors: We added the reasons of selecting study areas associate with  comparison of paddy farmer income and paddy productivity in provinces in Kalimantan in section Study area.

 Reviewer: and the clear way of selecting the sample from the population. Hence, I will give my recommendation after adding those comments.

 Authors: We added the random number method assigned every sample a number, then randomly picked samples by using a table of random numbers in section of Subject recruitment.

 Is the work clearly and accurately presented and does it cite the current literature?

 Reviewer: No

 Authors: Additional references in sections of Methods (Study area, Statistical analysis) and References.

 Is the study design appropriate and is the work tehnicaly sound?

 Reviewer: Partly

 Authors: Additional information in section of Methods (Study area and Subject recruitment).

 Are sufficient details of methods and analysis provided to allow replication by others?

 Reviewer: Partly

 Authors: Additional and revision of methods in sections of Methods (Statistical analysis) and Results and discussion (Tillage cost)

 If applicable, is the statistical analysis and its interpretation appropriate?

 Reviewer: Partly

 Authors: Additional the results of data analysis and information in section of Results and discussion (Characteristics of respondents, Hand tractor ownership, Hand tractor renter, and Tillage cost).

 Are all the source data underlying the results available to ensure full reproducibility?

 Reviewer: Partly

 Authors: The questionnaire and data already available in section of Data Availability (Underlying data  and Extended data).

 Are the conclusions drawn adequately supported by the results?

 Reviewer: Partly

 Authors: Additional and revision of analysis results in sections of  Conclusions and Abstract.

# Peer review: Reviewer response for version 1

**DOI:** 10.5256/f1000research.18576.r42260

**Published:** 2019-01-14

**Authors:** Ahmad Shuib

**Affiliations:** 1 University Putra Malaysia (UPM), Seri Kembangan, Malaysia

1. *Is the work clearly and accurately presented and does it cite the current literature?*
  Very limited references and a few dated references.
2. *Is the study design appropriate and is the work technically sound?*
  This is a straight forward descriptive study using Chi-squared tests to determine significant differences. It would have been more informative if the author had included the derivation of the costs of the equipment and the tillage operation from the user’s actual expenditure.
3. *Are sufficient details of methods and analysis provided to allow replication by others?*
  The process of collecting data was sufficiently discussed although the determination of the sample size was not clearly explained. If the data collection was carried out by the researcher herself without any assistant, it must have taken a long time to get data from 380 respondents. Samples of 1 and 2 for Bontang North and Bontang South respectively was not justified.
4. *If applicable, is the statistical analysis and its interpretation appropriate?*
  For a descriptive study to determine differences in usage of the machinery, the analysis is adequate. But for practical and policy decision purposes, the analysis may not provide sufficient evidence to help the relevant agencies to take the appropriate actions.
5. *Are all the source data underlying the results available to ensure full reproducibility?*
  The author has clearly discussed the sources of data used in the study.
6. *Are the conclusions drawn adequately supported by the results?*
  This is a simple descriptive study with straight forward conclusions. The analysis will be useful for further causal relationship analysis.

Is the work clearly and accurately presented and does it cite the current literature?

Partly

If applicable, is the statistical analysis and its interpretation appropriate?

Yes

Are all the source data underlying the results available to ensure full reproducibility?

Partly

Is the study design appropriate and is the work technically sound?

Yes

Are the conclusions drawn adequately supported by the results?

Yes

Are sufficient details of methods and analysis provided to allow replication by others?

Partly

Reviewer Expertise:

Natural Resource Economics, Tourism Planning and Development

I confirm that I have read this submission and believe that I have an appropriate level of expertise to confirm that it is of an acceptable scientific standard.

# Response

**Published:** 2021-04-10

**Authors:** Karmini Karmini

**Affiliations:** University of Mulawarman, Indonesia

**The authors revision for review on 14 January 2019 by Ahmad Shuib, University Putra Malaysia (UPM), Seri Kembangan, Malaysia.**

 1.  Is the work clearly and accurately presented and does it cite the current literature?

 Reviewer: Very limited references and  a few dated references.

 Authors: We added references of Pobhirun and Pinitsoontorn (2019), Statistics Indonesia (2008), Statistics East Kalimantan (2009), and Ogunbanwo et al. (2019) in section of References.

 2. Is the study design appropriate and is the work technically sound?

 Reviewer: This is a straight forward descriptive study using Chi-squared tests to determine significant differences.

 Authors: We added methods of Descriptive statistics (to count the frequency, total, percentage, maximum, and minimum) and One Way Anova  besides Chi Square One Sample (to test hypotheses) in section of Statistical analysis.

 Reviewer: It would have been more informative if the author had included the derivation of the costs of the equipment and the tillage operation from the user’s actual expenditure.

 Authors: We added information if the amount of hand tractor rental cost at the research location includes machine rental cost, machine maintenance cost, and operator wage in section of Tillage cost.

 3. Are sufficient details of methods and analysis provided to allow replication by others?

 Reviewer: The process of collecting data was sufficiently discussed although the determination of the sample size was not clearly explained.

 Authors: We added method to determine the sample size in section of Subject recruitment.

 Reviewer: If the data collection was carried out by the researcher herself without any assistant, it must have taken a long time to get data from 380 respondents.

 Authors: We added second author and enumerators whereas the researchers employed the enumerators to help the interviews process in sections of Author and Subject recruitment.

 Reviewer: Samples of 1 and 2 for Bontang North and Bontang South respectively was not justified.

 Authors: The determination of number of samples for North Bontang (24 paddy households; 1 sample) and South Bontang (120 paddy households; 2 samples) was already appropriate based on proportional sampling method and already fulfilled the minimum sample size for population (380 samples) in section of Subject recruitment.

 4. If applicable, is the statistical analysis and its interpretation appropriate?

 Reviewer: For a descriptive study to determine differences in usage of the machinery, the analysis is adequate. But for practical and policy decision purposes, the analysis may not provide sufficient evidence to help the relevant agencies to take the appropriate actions.

 Authors: We added recommendations if farm machinery are required  to develop paddy farming also Bontang and Penajam Paser Utara are the recommended regions for the activities in relation to increase the ownership of hand tractors because have higher tillage costs rather than Kutai Kartanegara in section of Conclusions.

 5. Are all the source data underlying the results available to ensure full reproducibility?

 Reviewer: The author has clearly discussed the sources of data used in the study.

 Authors: The questionnaire and data are already available in sections of Extended data and Underlying data

 6. Are the conclusions drawn adequately supported by the results?

 Reviewer: This is a simple descriptive study with straight forward conclusions.

 Authors: We revised the results of hypotheses testing in sections of Conclusions and Abstract.

 Reviewer: The analysis will be useful for further causal relationship analysis.

 Authors: We added some factors caused differences the number of hand tractor owners, the number of hand tractor renters, and the tillage costs among some regions in East Kalimantan, Indonesia in section of Conclusions.

 Is the work clearly and accurately presented and does it cite the current literature?

 Reviewer: Partly

 Authors: Additional references in section of References.

 Is the study design appropriate and is the work tehnicaly sound?

 Reviewer: Yes

 Authors: Additional information in sections of Methods (Statistical analysis) and Results and discussion (Tillage cost).

 Are sufficient details of methods and analysis provided to allow replication by others?

 Reviewer: Partly

 Authors: Additional of method, second author, and enumerators in sections of Author and Methods (Subject recruitment).

 If applicable, is the statistical analysis and its interpretation appropriate?

 Reviewer: Yes

 Authors: Additional recommendations in section of Conclusions.

 Are all the source data underlying the results available to ensure full reproducibility?

 Reviewer: Partly

 Authors: The questionnaire and data already available in section of Data Availability (Underlying data  and Extended data).

 Are the conclusions drawn adequately supported by the results?

 Reviewer: Yes

 Authors: Additional and revision of the analysis results in sections of Conclusions and Abstract.
